# Supplementary material for: HIVprotI: an integrated web based platform for prediction and design of HIV proteins inhibitors
Source: J Cheminform. 2018 Mar 9;10:12. doi: 10.1186/s13321-018-0266-y (PMC5845081; doi:10.1186/s13321-018-0266-y)
Supplement: Supplementary file 2 — Additional file 2. Source code of HIVProtI web server. [file 13321_2018_266_MOESM2_ESM.zip › HIVprotI_Source-code/hivprotI/weka-3-6-6/doc/weka/filters/unsupervised/attribute/NumericToNominal.html]

NumericToNominal


---


|  |  |  |
| --- | --- | --- |
| **Overview** \| **Package** \| **Class** \| **Tree** \| **Deprecated** \| **Index** \| **Help** \| **Weka's home** | |  |
| **PREV CLASS**   **NEXT CLASS** | **FRAMES**    **NO FRAMES**     **All Classes** |  |
| SUMMARY: NESTED \| FIELD \| CONSTR \| METHOD | DETAIL: FIELD \| CONSTR \| METHOD |  |


---


## weka.filters.unsupervised.attribute Class NumericToNominal

```
java.lang.Object
  weka.filters.Filter
      weka.filters.SimpleFilter
          weka.filters.SimpleBatchFilter
              weka.filters.unsupervised.attribute.NumericToNominal
```

**All Implemented Interfaces:**: java.io.Serializable, CapabilitiesHandler, OptionHandler, RevisionHandler

---

``` public class NumericToNominal extends SimpleBatchFilter ```

A filter for turning numeric attributes into nominal ones. Unlike discretization, it just takes all numeric values and adds them to the list of nominal values of that attribute. Useful after CSV imports, to enforce certain attributes to become nominal, e.g., the class attribute, containing values from 1 to 5.


Valid options are:

```
 -R <col1,col2-col4,...>
  Specifies list of columns to Discretize. First and last are valid indexes.
  (default: first-last)
```

```
 -V
  Invert matching sense of column indexes.
```

**Version:**
:   $Revision: 5543 $

**Author:**
:   fracpete (fracpete at waikato dot ac dot nz)

**See Also:**: Serialized Form

---

**Constructor Summary**

`NumericToNominal()`


| **Method Summary** | |
| --- | --- |
| `java.lang.String` | `attributeIndicesTipText()`             Returns the tip text for this property |
| `java.lang.String` | `getAttributeIndices()`             Gets the current range selection |
| `Capabilities` | `getCapabilities()`             Returns the Capabilities of this filter. |
| `boolean` | `getInvertSelection()`             Gets whether the supplied columns are to be worked on or the others. |
| `java.lang.String[]` | `getOptions()`             Gets the current settings of the filter. |
| `java.lang.String` | `getRevision()`             Returns the revision string. |
| `java.lang.String` | `globalInfo()`             Returns a string describing this filter |
| `java.lang.String` | `invertSelectionTipText()`             Returns the tip text for this property |
| `java.util.Enumeration` | `listOptions()`             Gets an enumeration describing the available options. |
| `static void` | `main(java.lang.String[] args)`             Runs the filter with the given parameters. |
| `void` | `setAttributeIndices(java.lang.String value)`             Sets which attributes are to be "nominalized" (only numeric attributes among the selection will be transformed). |
| `void` | `setAttributeIndicesArray(int[] value)`             Sets which attributes are to be transoformed to nominal. |
| `void` | `setInvertSelection(boolean value)`             Sets whether selected columns should be worked on or all the others apart from these. |
| `void` | `setOptions(java.lang.String[] options)`             Parses a given list of options. |

**Methods inherited from class weka.filters.SimpleBatchFilter**

`batchFinished, input`

**Methods inherited from class weka.filters.SimpleFilter**

`debugTipText, getDebug, setDebug, setInputFormat`

**Methods inherited from class weka.filters.Filter**

`batchFilterFile, filterFile, getCapabilities, getOutputFormat, isFirstBatchDone, isNewBatch, isOutputFormatDefined, makeCopies, makeCopy, numPendingOutput, output, outputPeek, toString, useFilter, wekaStaticWrapper`

**Methods inherited from class java.lang.Object**

`equals, getClass, hashCode, notify, notifyAll, wait, wait, wait`

**Constructor Detail**

### NumericToNominal

```
public NumericToNominal()
```


**Method Detail**

### globalInfo

```
public java.lang.String globalInfo()
```

:   Returns a string describing this filter

    :   **Specified by:**: `globalInfo` in class `SimpleFilter`
    :   **Returns:**: a description of the filter suitable for displaying in the explorer/experimenter gui

---


### listOptions

```
public java.util.Enumeration listOptions()
```

:   Gets an enumeration describing the available options.

    :   **Specified by:**: `listOptions` in interface `OptionHandler` **Overrides:**: `listOptions` in class `SimpleFilter`
    :   **Returns:**: an enumeration of all the available options.

---


### setOptions

```
public void setOptions(java.lang.String[] options)
                throws java.lang.Exception
```

:   Parses a given list of options.


    Valid options are:

    ```
     -R <col1,col2-col4,...>
      Specifies list of columns to Discretize. First and last are valid indexes.
      (default: first-last)
    ```

    ```
     -V
      Invert matching sense of column indexes.
    ```

    :   **Specified by:**: `setOptions` in interface `OptionHandler` **Overrides:**: `setOptions` in class `SimpleFilter`
    :   **Parameters:**: `options` - the list of options as an array of strings **Throws:**: `java.lang.Exception` - if an option is not supported **See Also:**: `SimpleFilter.reset()`

---


### getOptions

```
public java.lang.String[] getOptions()
```

:   Gets the current settings of the filter.

    :   **Specified by:**: `getOptions` in interface `OptionHandler` **Overrides:**: `getOptions` in class `SimpleFilter`
    :   **Returns:**: an array of strings suitable for passing to setOptions

---


### invertSelectionTipText

```
public java.lang.String invertSelectionTipText()
```

:   Returns the tip text for this property

    :   **Returns:**: tip text for this property suitable for displaying in the explorer/experimenter gui

---


### getInvertSelection

```
public boolean getInvertSelection()
```

:   Gets whether the supplied columns are to be worked on or the others.

    :   **Returns:**: true if the supplied columns will be worked on

---


### setInvertSelection

```
public void setInvertSelection(boolean value)
```

:   Sets whether selected columns should be worked on or all the others apart
    from these. If true all the other columns are considered for
    "nominalization".

    :   **Parameters:**: `value` - the new invert setting

---


### attributeIndicesTipText

```
public java.lang.String attributeIndicesTipText()
```

:   Returns the tip text for this property

    :   **Returns:**: tip text for this property suitable for displaying in the explorer/experimenter gui

---


### getAttributeIndices

```
public java.lang.String getAttributeIndices()
```

:   Gets the current range selection

    :   **Returns:**: a string containing a comma separated list of ranges

---


### setAttributeIndices

```
public void setAttributeIndices(java.lang.String value)
```

:   Sets which attributes are to be "nominalized" (only numeric
    attributes among the selection will be transformed).

    :   **Parameters:**: `value` - a string representing the list of attributes. Since the string will typically come from a user, attributes are indexed from 1. eg: first-3,5,6-last **Throws:**: `java.lang.IllegalArgumentException` - if an invalid range list is supplied

---


### setAttributeIndicesArray

```
public void setAttributeIndicesArray(int[] value)
```

:   Sets which attributes are to be transoformed to nominal. (only numeric
    attributes among the selection will be transformed).

    :   **Parameters:**: `value` - an array containing indexes of attributes to nominalize. Since the array will typically come from a program, attributes are indexed from 0. **Throws:**: `java.lang.IllegalArgumentException` - if an invalid set of ranges is supplied

---


### getCapabilities

```
public Capabilities getCapabilities()
```

:   Returns the Capabilities of this filter.

    :   **Specified by:**: `getCapabilities` in interface `CapabilitiesHandler` **Overrides:**: `getCapabilities` in class `Filter`
    :   **Returns:**: the capabilities of this object **See Also:**: `Capabilities`

---


### getRevision

```
public java.lang.String getRevision()
```

:   Returns the revision string.

    :   **Specified by:**: `getRevision` in interface `RevisionHandler` **Overrides:**: `getRevision` in class `Filter`
    :   **Returns:**: the revision

---


### main

```
public static void main(java.lang.String[] args)
```

:   Runs the filter with the given parameters. Use -h to list options.

    :   **Parameters:**: `args` - the commandline options


---


|  |  |  |
| --- | --- | --- |
| **Overview** \| **Package** \| **Class** \| **Tree** \| **Deprecated** \| **Index** \| **Help** \| **Weka's home** | |  |
| **PREV CLASS**   **NEXT CLASS** | **FRAMES**    **NO FRAMES**     **All Classes** |  |
| SUMMARY: NESTED \| FIELD \| CONSTR \| METHOD | DETAIL: FIELD \| CONSTR \| METHOD |  |


---
